# Revealing the thermal oxidation stability and its mechanism of rice bran oil

**DOI:** 10.1038/s41598-020-71020-y

**Published:** 2020-08-24

**Authors:** Halida Rahmania, Shunji Kato, Kazue Sawada, Chieko Hayashi, Hiroyuki Hashimoto, Shigeo Nakajima, Yurika Otoki, Junya Ito, Kiyotaka Nakagawa

**Affiliations:** 1 Food and Biodynamic Chemistry Laboratory, Graduate School of Agricultural Science, Tohoku University, Sendai, Miyagi 980-8572 Japan; 2 Tsuno Food Industrial Co., Ltd., Ito, Wakayama 649-7194 Japan

**Keywords:** Quality of life, Health care

## Abstract

Although the stability of rice bran oil (RBO) has been showed on several studies, the factors which make it capable on maintaining its stability under thermal oxidation has not been sure yet. We hypothesized that its fatty acid composition [high composition of oleic acid (OA), lower composition of linoleic acid (LA) and α-linolenic acid (LnA)] and/or its antioxidant agents [γ-oryzanol (OZ)] and vitamin E [tocopherol (Toc), tocotrienol (T3)] might be the biggest factor. To prove the hypothesis, we thermally oxidized RBO under 40 °C for 17 days to mimic the harsh daily storage condition, and compared it with soybean oil (SO) and rapeseed oil (RPO) then monitoring their primary oxidation products [triacylglycerol hydroperoxide (TGOOH)] from easily oxidized fatty acid contained in triacylglycerol (TG) and the amount loss of antioxidant agents. As a result, RBO showed the lowest TGOOH/TG ratio, followed by RPO and SO. The superior stability RPO compared SO might occur due to because of the influence of the fatty acid profile (higher OA and lower LA). For RBO’s case, besides its fatty acid profile, the existence of OZ and the synergistic effect of OZ and vitamin E might have a greater contribution in maintaining its stability under thermal oxidation.

## Introduction

One of the best things about rice bran oil (RBO), an oil extracted from the bran layer of the rice kernel, would be its stability, which secures the value of RBO and its related products^1–3^. Indeed, some studies have shown the higher thermal-oxidation stability of RBO compared to other edible oils^4–7^, nevertheless the mechanism of the stability of RBO is still speculative. Its fatty acid profile and/or its antioxidant agents might be one of the biggest reasons; namely, contents of easily oxidized fatty acids [i.e. poly-unsaturated fatty acid such as linoleic acid (LA) and α-linolenic acid (LnA)], stable fatty acid [i.e. mono-unsaturated fatty acid such as oleic acid (OA)] and/or antioxidants [tocopherol (Toc), tocotrienol (T3), and γ-oryzanol (OZ)] in RBO have been hypothesized as possible factors that made RBO showed a stability under thermal oxidation^8–13^.

However, verification of the above hypothesis has been severely hampered, because of the fact that the common methods used to determine the oils’ stability [e.g., peroxide value (POV), rancimat method, and spectrophotometry] cannot provide enough information, especially about which and how much fatty acids are actually oxidized^14^ and whether the antioxidant agents can affect/improve RBO’s stability during the thermal oxidation^13^. Overcoming this issue will lead to developing more quality and confident RBO and its related products.

**Figure 1:**
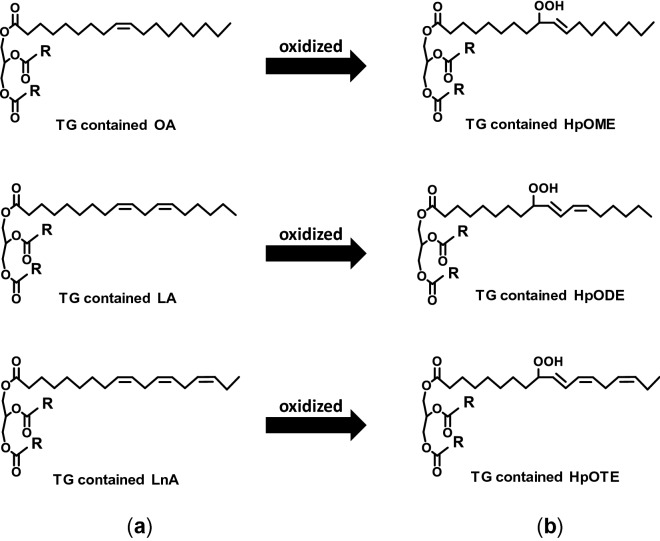
The chemical structure of selected TG (**a**) and its TGOOH products (**b**).

In this study, for evaluating the contribution of individual fatty acids of RBO, we explored the use of the two-dimensional (2D) profiling method with liquid chromatography time-of-flight mass spectrometry (LC-TOF/MS)^15^ to determine the most suitable target of triacylglycerol (TG) contained in fresh RBO which structurally composed either OA, LA or LnA in one of fatty acid composition of TG (Fig. 1a). Next, we treated the oil under thermal conditions and examined the TG hydroperoxide (TGOOH) products (i.e. TGOOH isomers) as primary oxidation products that specifically formed from the target TG (Fig. 1b). Such TGOOH isomers were high-selectively identified by our analytical method for lipid hydroperoxides with liquid chromatography tandem mass spectrometry (LC–MS/MS)^14,16–19^. Besides RBO, we measured and compared the target TG and TGOOH isomers in the oil with soybean oil (SO) and rapeseed oil (RPO) as those edible oils also have a different unique fatty acid profile^20^.

As mentioned in the previous studies, it has been reported that antioxidant agents such as OZ, Toc, and T3 have a great antioxidant activity. This might also be the reason why RBO obtained its ability to be stable under thermal oxidation. To evaluate the contribution of antioxidant agents, the content of antioxidants agents in RBO and other edible oils (SO and RPO) was examined by comparing the loss of the antioxidant contents during the thermal oxidation^2,21–24^.

In the field of oil chemistry, since most studies usually consider the data of POV, rancimat method, and/or spectrophotometry for evaluating the oils’ stability, to the best of our knowledge, no approaches like this study have been carried out so far. The findings from this study also would provide the comprehensive understanding on the contribution of fatty acids and antioxidant agents contained in the RBO on its stability under the thermal condition. These knowledges are anticipated to be beneficial in finding the best manner to keep (possibly to further improve) the oil stability, not only for RBO, but also for other edible oils and their related products.

## Results and discussion

### Hypothesis about fatty acid and antioxidant agent theory

As described in Introduction, the higher thermal-oxidation stability is the distinctive character of RBO^1–7^, although the mechanism is still unclear. It is believed that the RBO’s fatty acid profile and/or its antioxidant agents would be one of the biggest reasons for the stability^8–13^.

To evaluate the role of fatty acids during the thermal oxidation, we investigated the contribution of specific fatty acid (i.e., OA, LA, and LnA) contained in RBO towards its thermal oxidation stability. Specifically, we first determined the TG specific target which bearing at least one of either OA, LA, or LnA and then monitored their specific primary oxidation products (TGOOH). To evaluate the hypothesis about the antioxidant agent theory, the content of antioxidants in RBO and other edible oils was examined by comparing the loss of the antioxidant contents during the thermal oxidation.

### LC–TOF/MS determination of TG target

TG consists of a glycerol backbone and three various fatty acids [e.g. RBO: palmitic acid (PA) 17.42%, stearic acid (SA) 1.58%, OA 43.06%, LA 35.90% and LnA 0.86%; Supplementary Table S1 online], which gives a lot variety of TG species. To find out the most suitable TG target, comprehensive TG analysis was first performed.

**Figure 2:**
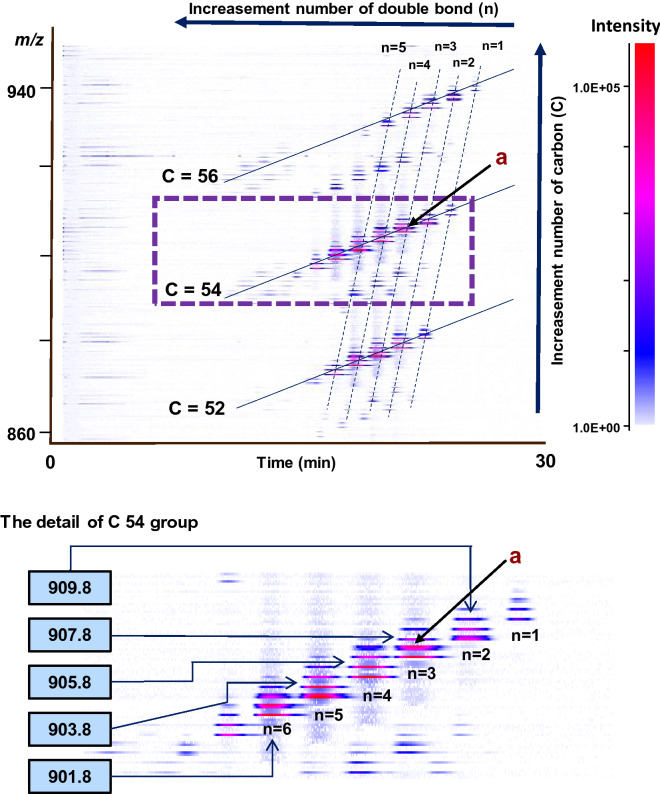
The chromatogram of triacylglycerol (TG) contained in fresh RBO analyzed by using LC-TOF/MS (ESI (+)). Fresh RBO was dissolved 100-fold volume of isopropanol then 100-fold volume of methanol. MS spectra were obtained in the *m/z* ranges of 500–950. The 2D profiling was constructed with X (retention time) and Y (*m/z* value) axes, and abundance of individual TAGs was indicated by single color density (originally adjusted by color gradient). The broken line showed the group of the same number of double bond while the solid line showed the group of the same number of carbons. The 2D profiling of fresh RBO created in this figure was generated by using Daltonics DataAnalysis (ver. 4.1) under Bruker Daltonik GmbH license (https://www.bdal.com).

Recently, Ikeda et al. reported that the comprehensive TG analysis in mouse liver and white adipose tissue by using 2D profiling^15^. Briefly, in the reverse-phase-LC, TG species are generally eluted depending on the number of carbon and double bond of fatty acid in TG [e.g., as the number of carbon (double bond) is increased (decreased), TG elution time is late]. Therefore, TG peaks were separated and distributed in order on the 2D profiling based on the number of carbon and double bond. By adapting the same method, we explored the comprehensive TG analysis consisted in RBO. Figure 2 shows a 2D profiling of fresh RBO (x-axis: retention time, y-axis: *m/z*). As shown in 2D profiling, TG species were located in certain lines based on the structure. To assign each line, the OA-OA-OA standard (C = 54, n = 3) was analyzed. The line on RBO's TG 2D profiling data (point a) which was in the same position as the OA-OA-OA standard was considered to contain C = 54 and n = 3. Therefore, the other line on the 2D profiling could be assigned as well [C = 52, 54 and 56 (solid lines) and n = 1–6 (broken lines)]. As a result, RBO was found to be abundant in TG species composed of C = 54 which contained n = 3–6 (*m/z* 907.8, *m/z* 905.8, *m/z* 903.8, and *m/z* 901.8 [M + Na]^+^). Considering the low amount of SA (1.6%) in RBO, TG species on the C = 54, n = 3 (*m/z* 907.8) would possess at least one of OA. TG on the C = 54, n = 4 (*m/z* 905.8) should possess at least one of LA. In addition, LnA was considered to be exist in the TG species bearing more of the double bonds [C = 54, n ≥ 5 (*m/z* 903.8, and *m/z* 901.8)]. Accordingly, the TG species of “C = 54 and n ≥ 3” were considered to be suitable to evaluate the contribution of specific fatty acid (i.e., OA, LA, and LnA) on oil oxidation.

**Figure 3:**
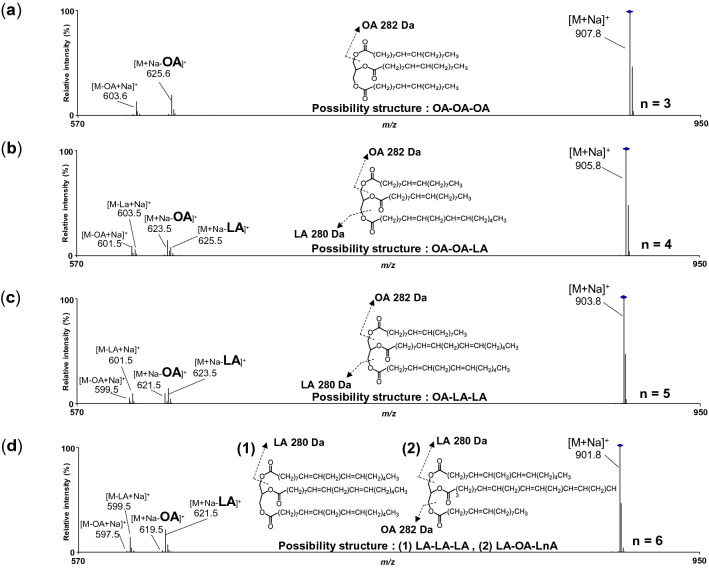
The product ion of TG *m/z* 907.8 [M + Na]^+^ (**a**), 905.8 [M + Na]^+^ (**b**), 903.8 [M + Na]^+^ (**c**), and 901.8 [M + Na]^+^ (**d**) by using LC-TOF/MS (ESI (+)). Fresh RBO was dissolved in 100-fold volume of isopropanol then 100-fold volume of methanol. The 2 mM of sodium acetate in methanol was used as post column additive to promote the ionization. Insets show the speculated fragmentation patterns of TG species.

**Figure 4:**
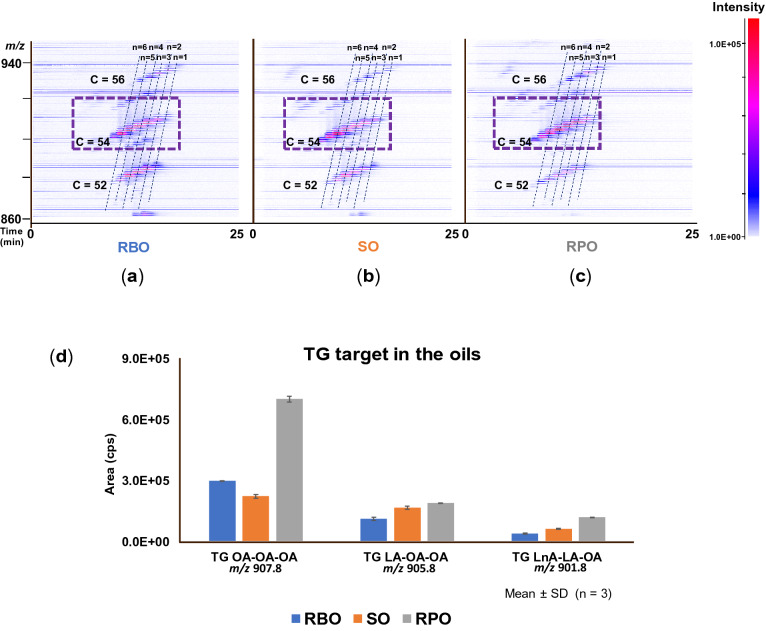
The chromatogram of triacylglycerol (TG) contained in fresh RBO (**a**), SO (**b**), and RPO (**c**) analyzed by using LC-TOF/MS with ESI (+). The fresh oils were dissolved 100-fold volume of isopropanol then 100-fold volume of methanol. MS spectra were obtained in the *m/z* ranges of 500–950. The 2D profiling was constructed with X (retention time) and Y (*m/z* value) axes, and abundance of individual TAGs was indicated by single color density (originally adjusted by color gradient). The broken line showed the group of the same number of double bonds. The amount of TG targets (*m/z* 907.8; *m/z* 905.8, and *m/z* 901.8) contained in each oils were also calculated (**d**). The 2D profiling of fresh RBO (**a**), SO (**b**), and RPO (**c**) created in this figure were generated by using Daltonics DataAnalysis (ver. 4.1) under Bruker Daltonik GmbH license (https://www.bdal.com).

To further explore the TG species of “C = 54 and n ≥ 3” configuration, we conducted the product ion scan to analyze the detailed fatty acid composition. When TG (*m/z* 907.8) was analyzed as a precursor ion, fragment ion *m/z* 625.6 and *m/z* 603.6 were abundantly detected (Fig. 3a). These ions were considered to be derived from neutral loss (NL) of oleoyl moiety (OA, − 282 Da) (*m/z* 603.6 was de-sodiated ion of *m/z* 625.6). Since any other fragment ions were not detected, TG (*m/z* 907.8) should be composed of only OA, which coincides with 2D profiling (C = 54 and n = 3). Accordingly, TG (*m/z* 907.8) was found to be OA-OA-OA. Similarly, the TG (*m/z* 905.8) generated the fragment ions as follows; *m/z* 623.5 (NL of OA), *m/z* 625.5 (NL of LA), *m/z* 601.5 (*m/z* 623.5–Na^+^) and *m/z* 603.5 (*m/z* 625.5–Na^+^) in abundance (Fig. 3b). Considering the 2D profiling (C = 54, n = 4), TG (*m/z* 905.8) was considered to be OA-OA-LA. Similarly, TG (*m/z* 903.8, C = 54, n = 5) was also determined as OA-LA-LA (Fig. 3c). TG (*m/z* 901.8, C = 54, n = 6) generated mainly *m/z* 621.5 and *m/z* 599.5 (*m/z* 621.5–Na^+^), suggesting that almost TG (*m/z* 901.8) is composed of LA-LA-LA (Fig. 3d). On the other hand, little *m/z* 619.5 (NL of OA) was also detected, suggesting the presence of TG bearing OA. Since the total number of the double bond of TG (*m/z* 901.8) is six, residual double bonds (i.e., n = 5) are allocated to two fatty acids. Because of the absence of fatty acid (n = 4 and 5), remaining fatty acids should be LA and LnA. However, distinct NL of LnA could not be observed in the mass spectrum. The reason was considered that isotope of *m/z* 621.5 (NL of LA) would be overlapped with the fragment derived from NL of LnA. Accordingly, we considered that TG (*m/z* 901.8) also contains OA-LA-LnA in addition to LA-LA-LA. Those TG species (*m/z* 907.8, *m/z* 905.8, *m/z* 903.8, and *m/z* 901.8) had also been detected abundantly in SO and RPO (Fig. 4). Considering the TG abundance in RBO and also in the other oils (SO and RPO); and the fatty acid contained in the TG composition, therefore, we decided to choose OA-OA-OA (*m/z* 907.8), OA-OA-LA (*m/z* 905.8), and OA-LA-LnA (*m/z* 901.8) as TG target to evaluate the contribution of fatty acids (OA, LA, and LnA) on RBO stability under thermal oxidation.

### LC–MS/MS analysis of TGOOH

**Figure 5:**
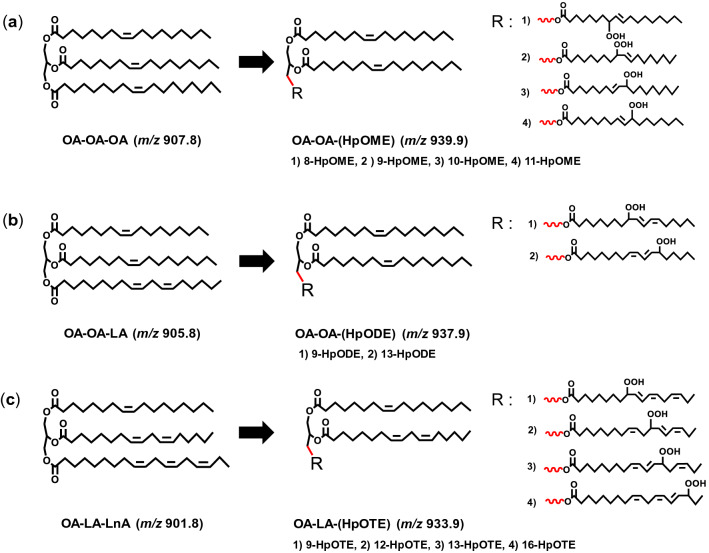
The TGOOH products from selected TG; OA-OA-OA produced OA-OA-HpOME (**a**), OA-OA-LA produced OA-OA-HpODE (**b**), and OA-LA-LnA produced OA-LA-HpOTE (**c**) The *) showed the isomers of each TGOOH products.

Under thermal oxidation, the TG target will convert to its specific TGOOH products; OA-OA-(8-hydroperoxy octadecamonoenoyl (HpOME)); OA-OA-(9-HpOME), OA-OA-(10-HpOME) and OA-OA-(11-HpOME) (Fig. 5a); OA-OA-(9-hydroperoxy octadecadienoyl (HpODE)), and OA-OA-(13-HpODE) (Fig. 5b); and OA-LA-(9-hydroperoxy octadecatrienoyl (HpOTE)), OA-LA-(12-HpOTE), OA-LA-(13-HpOTE), and OA-LA-(16-HpOTE) (Fig. 5c)^25^. To distinguish the each TGOOH isomers, we referred to our previous LC–MS/MS studies that have successfully separated lipid hydroperoxide isomers by unique fragmentation derived from sodiated form^13,20–23^. This fragmentation is considered to occur near the hydroperoxyl group by α-cleavage or Hock fragmentation^26^. We had already been successful in analysis of OA-OA-HpODE isomers in RPO by using this fragmentation^13^. Accordingly, we expanded the use of the same method to determine the fragmentation not only for OA-OA-HpODE, but also OA-OA-HpOME and OA-LA-HpOTE.

**Figure 6:**
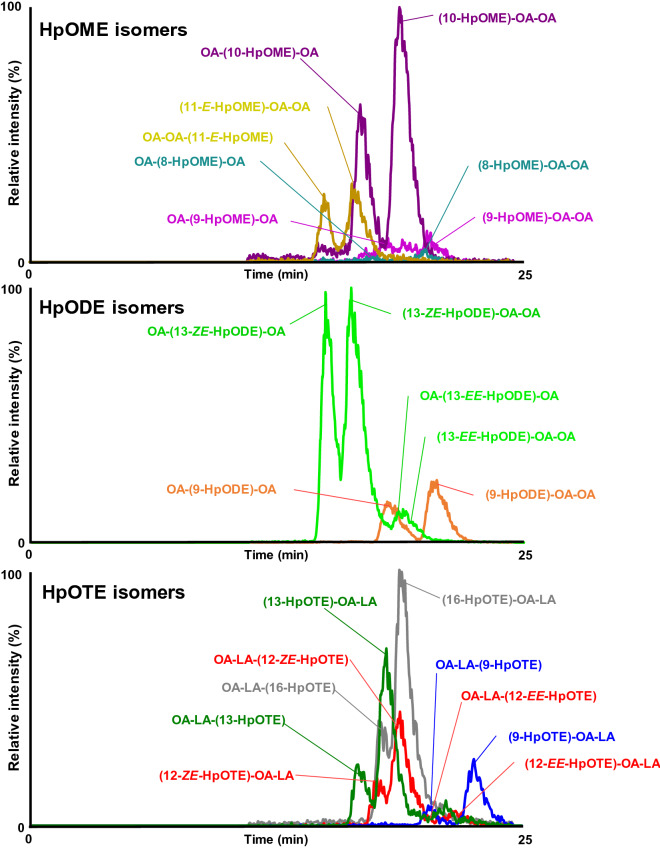
The chromatogram of primary oxidation product formed in RBO thermally oxidized (40 °C, 17 days, dark condition). The oils were diluted 100-fold by using hexane and approximately 10 μL was subjected into LC–MS/MS with ESI (+). A solution containing sodium acetate (0.2 mM) was used to promote the ionization. MRM was paired to distinguish the TGOOH isomers.

During the thermal oxidation, LA will be oxidized and generate two HpODE isomers that can be identified based on their unique NL during the collision (i.e., NL 169 Da for isomer 9-HpODE and 88 Da for 13-HpODE)^14^. Similar concept was used to analyze the oxidation product of OA (HpOME isomers) and LnA (HpOTE isomers). In case of the oxidation of OA, it generates four HpOME isomers (i.e. 185 Da for isomer of 8-HpOME, 171 Da for 9-HpOME, 130 Da for 10-HpOME, and 116 Da for 11-HpOME), while oxidation of LnA generates four isomers (i.e., 167 Da for 9-HpOTE, 127 Da for 12-HpOTE, 86 Da for 13-HpOTE, and 46 Da for 16-HpOTE), respectively. Based on the characteristic of fragment ions derived from the NL of product ion, we used MRM to distinguish each isomer. As we expected, in the preliminary analysis which analyzed fresh and oxidized RBO (17 days) (Fig. 6), the increment of each TGOOH isomers was actually observed, suggesting that multiple reaction monitoring (MRM) pairs used in this study are highly useful for the evaluation of oil oxidation under the thermal condition.

### Evaluation of RBO, SO and RPO stability using POV and LC–MS/MS method

**Figure 7:**
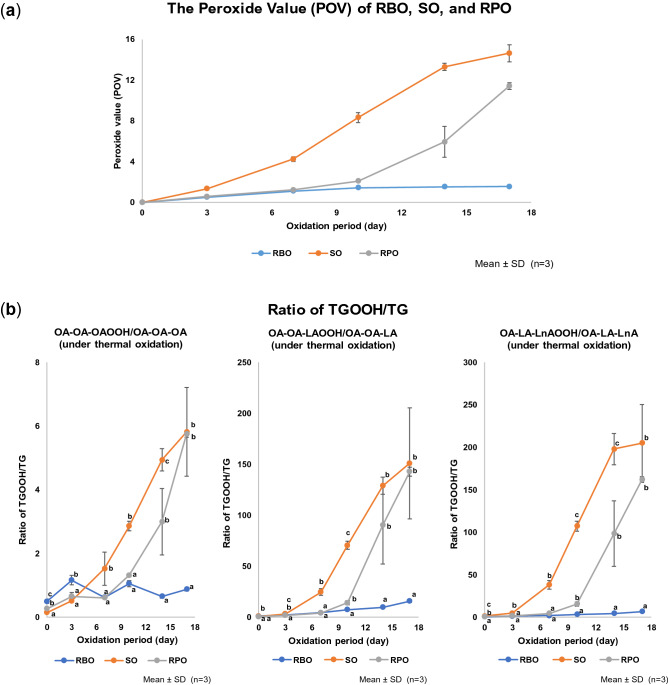
The peroxide value (POV) (**a**) and ratio of TGOOH/TG products (**b**) of RBO, SO, and RPO under thermal oxidation (at 40 °C for 17 days of oxidation period, under dark condition). Values are expressed as mean ± SD, n = 3. The POV was determined by titration method, meanwhile the ratio of TGOOH/TG was determined by LC–MS/MS. The different alphabets shown in graphic (**b**) means that the group on the same oxidation period has significantly different (*p* < 0.05).

Stability evaluation on the oils was firstly determined by using common method (POV), determined by iodometric titration, which has been shown on Fig. 7a. In accordance with previous studies^4–7^, RBO showed the best stability under thermal oxidation compared to other oils, followed by RPO and SO.

To find out the contribution of fatty acids on RBO stability, TGOOH analysis was then performed. In this study, we summed the peak area of each OA-OA-(HpOME) isomer (i.e., OA-OA-(8-HpOME), OA-OA-(9-HpOME), OA-OA-(10-HpOME) and OA-OA-(11-HpOME)), OA-OA-(HpODE) isomer (i.e., OA-OA-(9-HpODE) and OA-OA-(13-HpODE)) and OA-LA-(HpOTE) isomer (i.e., OA-LA-(9-HpOTE), OA-LA-(12-HpOTE), OA-LA-(13-HpOTE) and OA-LA-(16-HpOTE)). In addition, the ratio of TGOOH to its corresponding TG (e.g., OA-OA-(HpOME) to OA-OA-OA) was calculated to compare the RBO oxidation with SO and RPO. The graphic showed the ratio of TGOOH/TG (area per area) from each fatty acid could be seen in Fig. 7b. Based on the TGOOH/TG ratio, RBO showed the best stability against thermal oxidation among the three oil types (or compared to SO and RPO). Interestingly, the curves of TGOOH/TG ratio also demonstrated the high positive correlation with a POV curve in all TGOOH. In regards to SO, the TGOOH/TG ratio curve for OA-LA-LnA reached the plateau at earlier time point (day 14) compared to OA-OA-OA and OA-OA-LA, thus indicated the preferential oxidation of OA-LA-LnA. On the contrary, in RBO, slight increase in the oxidation of OA-OA-LA and OA-LA-LnA were observed, demonstrating early stage of the oxidation process. Overall, our results signify the superiority (or advantage) of our method in providing more detailed information on the oxidation profile in oil when compared to POV method.

### Effect of fatty acid composition and antioxidant components on the stability of RBO, SO, and RPO

Previous studies have reported that similar fatty acids species possess similar relative oxidation rate^27,28^ that should be also reflected in the TGOOH/TG curves, unless there are effects from oil matrix such as fatty acid profile or antioxidants. In this study, longer lag-phase were observed in RPO and RBO compared with SO (Fig. 7b), despite the higher amount of vitamin E in SO rather than RPO and RBO (Supplementary Table S2 online). This would indicate that other internal factors (i.e., fatty acid and/or other antioxidants) also contribute to this phenomenon. For instance, the relative RPO stability compared with SO might be explained by the high amount of OA and low amount of LA^8–12^. On the other hand, despite the OA content in RBO was lower than RPO and the LA content was also higher (Supplementary Table S1 online), RBO still showed the incredible stability, much better than RPO. From these results, it was considered that the antioxidant agents contained in RBO might have bigger contribution in maintaining RBO stability in comparison to fatty acid profile.

Accordingly, to evaluate the antioxidant effect, antioxidants (i.e., OZ, Toc and T3) were analyzed (Toc and T3 were summed as vitamin E). Vitamin E, known as free radical scavenger^23^, in RBO mostly did not decrease during 17 days, while those of SO and RPO clearly decreased (Supplementary Fig. S1a online). In addition to vitamin E, OZ also did not decrease in RBO (not detected in SO and RPO) in accordance with previous study (Supplementary Fig. S1b online)^29^. The previous studies have mentioned that the antioxidant activity of OZ is higher than 4 isomers of vitamin E (α-, γ- of Toc and T3) and OZ is the main antioxidant agent in RBO^24,30^. In addition to such antioxidant activity of OZ itself, OZ also seems to affect to vitamin E stabilization, as the amount of vitamin E in RBO was stable during the thermal condition. It has been reported that OZ might protect Toc from degradation since it has successfully suppressed the rate of Toc degradation in dough fried in soybean oil contained rice bran oil^31^. The other study has also mentioned the synergic effect of 1% OZ (80% purity) and 0.1% α-Toc contained in sunflower oil^22^. Taken together, the existence of OZ and the synergic activity of OZ and vitamin E might be the major reason that support the RBO stability under thermal conditions.

## Conclusions

In this study, we analyzed the contribution of fatty acid profile and antioxidants on RBO stability as for giving an overview on the oil’s stability under extreme long-shelf storage time (thermal oxidation stability). Especially, to get the insight about the fatty acid contribution, 2D profiling and unique fragmentation by sodium ion were combined and TGOOH were analyzed in RBO, SO and RPO. As a result, it was suggested that the fatty acid profile in the oil (e.g., high amount of OA and/or low amount of LA and LnA) might have a contribution on the oil stability under thermal condition. However, even so, the stability of RBO was remarkable compared to the other oils. The stability of RBO also might be highly affected by the presence of OZ and synergistic effect of OZ and vitamin E. Even this study has provided some insight about RBO’s oxidation stability mechanism under thermal condition, but whether the RBO maintains its stability under photo oxidation remains unclear. As the previous study mentioned, the oxidation mechanism of photo oxidation is different from the thermal oxidation^25^, and several different things might affect the oxidation mechanism that occurs in RBO during photo oxidation. The photo oxidation study is also important to identify as for giving an overview of possibility light exposure during storage time. Therefore, the study of the oxidation stability mechanism of RBO under the photo-oxidation will be further examined to lead to further improvement of the RBO's quality.

## Materials and methods

### Materials

RBO, SO, and RPO were received from Tsuno Food Industrial Co., Ltd (Wakayama, Japan). The fatty acid composition in the oils (RBO, SO, and RPO) was evaluated by gas chromatography (GC) method^14^. A typical OZ, cycloartenyl ferulate, was purchased from FUJIFILM Wako Pure Chemical Corp. (Osaka, Japan). α, β, γ and δ-Toc were obtained from Merck KGaA (Darmstadt, Germany). α, β, γ and δ-T3 were from Cayman Chemical Co., Inc (Michigan, USA). Unless otherwise noted, all other reagents had been purchased from FUJIFILM Wako Pure Chemical Corp., and are the highest grade available.

### Thermal oxidation of RBO, SO and RPO

Our study’s purpose is to evaluate the oils’ stability under thermal oxidation. The temperature 40 °C was chosen as expected to be the harsh storage condition in daily life (i.e. mid-summer). The oxidation period was carried out for 17 days in order to give an overview of a long storage time.

Twenty grams of each RBO, SO and RPO was dispensed in the clear glass bottle (PS-4 K, Daiichi Glass Co., Ltd., Tokyo, Japan), tightly closed, and placed inside the oven with the temperature set at 40 °C under the dark condition for 17 days. The oil samples were taken at 0 (fresh oil), 3, 7, 10, 14 and 17 days (n = 3) and stored at − 30 °C until analysis. Prior to storage, nitrogen gas was flown inside the sample bottle to prevent further oxidation.

### LC-TOF/MS determination of TG target

The previous study has mentioned the successful use of 2D profiling method to analyze TG structure with detailed information (i.e. the number of carbon and double bond) in biological samples by using LC-TOF/MS^15^. As mentioned before, TG targets will be used for evaluating the contribution of fatty acids (OA, LA, and LnA) during thermal oxidation. In this study, we utilized the basic concept to determine the TG target in fresh RBO sample with some modifications (e.g., LC and MS conditions). The TG targets that we looked for should contain either OA, LA, or LnA in one of its fatty acid compositions and could be abundantly found in RBO. After determining the TG targets from RBO, we then confirmed the existence of such TG targets in SO and RPO. The analysis was performed as described below.

The oil samples were diluted 100-fold using 2-propanol, then diluted again 100-fold using methanol. The solution (10 µL) was injected into the LC system (Shimadzu Corp., Kyoto, Japan). The column used was COSMOSIL 5C18-MS-II, 5 µm, 2.0 ID × 250 mm (Nacalai Tesque, Inc., Kyoto, Japan) with a binary gradient consisting of solvent A (methanol) and solvent B (2-propanol). The gradient profile was as follows: 0–35 min, 35–100% B linear. The flow rate was 0.2 mL/min, and the column temperature was 40 °C. The column eluent was mixed with a post-column solvent consisting of methanol containing 2 mM sodium acetate, at 0.01 mL/min, to promote ionization. The combined flow was sent to TOF/MS (time-of-flight mass spectrometry, micrOTOF-Q II, Bruker Daltonics GmbH, Bremen, Germany) with positive electrospray ionization (ESI). MS was performed in the *m/z* range 500–950. The MS parameters are shown in Supplementary Table S3 online, which were optimized by OTOF Control Software (ver. 3.2). 2D profiling maps were constructed by using software Daltonics DataAnalysis (ver. 4.1).

### LC–MS/MS analysis of TGOOH

Our previous LC–MS/MS studies had extensively demonstrated the use of sodium ion to induce collision near the lipid hydroperoxides, which enabled distinctive identification of each TGOOH isomers^14,16–19^. In this study we expanded the use of the method to distinguish the TGOOH bearing OA, LA, and LnA hydroperoxide formed in the oxidized oils. Instrumental parameters were optimized by using oxidized RBO prepared as described above (17 days), and then all other samples were analyzed. Analysis were performed as described below.

The oil samples were diluted 100-fold using hexane. The solution (10 µL) was injected into the LC system (ExionLC, AB Sciex Pte., Ltd., Tokyo, Japan). The column used was Inertsil SIL-100A, 5 µm, 2.1 ID × 250 mm (GL Sciences Inc., Tokyo, Japan). Hexane–2-propanol–acetic acid (1,000:6:1, v/v/v) was used as a mobile phase with flow rate 0.2 mL/min. The post-column solution (methanol–2-propanol (1:1, v/v, 0.2 mM sodium acetate)) was mixed to the elution at 0.2 mL/min to promote ionization. The TGOOH was analyzed by triple quadrupole mass spectrometer (4000 QTRAP, SCIEX, Tokyo, Japan) with positive ESI. The expected MRM pairs were calculated based on the specific NL of each TGOOH isomers. The MS parameter [collision energy (CE) and collision cell exit potential (CXP)] were optimized for each TGOOH isomers (Supplementary Table S4 online) according to the Analyst software (ver. 1.6.2).

### POV measurement

The POV of the oxidized RBO, SO, and RPO was evaluated according to Japan Oil Chemists’ Society (JOCS) 2.5.2^32^.

### Determination of OZ, Toc, and T3

For OZ analysis, the RBO was diluted with 2-propanol (10 mg/mL) and subjected to LC-UV (320 nm). The OZ species (e.g., cycloartenyl ferulate, 24-methylenecycloartanyl ferulate, cyclosadyl ferulate, campesteryl ferulate, cyclobranyl ferulate, and β-sitosteryl ferulate) were separated by using Cadenza CD-C18, 3 μm, 3.0 ID × 250 mm (Imtakt Corp., Kyoto, Japan) utilized with Cadenza CD-C18, 3 μm, 2.0 ID × 5.0 mm as guard column which has set at 40 °C. A solution contained methanol–acetic acid (99:1, v/v) was used as a mobile phase with flow rate 0.45 mL/min. OZ concentration was calculated by using the standard curve of cycloartenyl ferulate (1.2–20.2 ng).

For Toc and T3 analysis, the sample was prepared by dissolving each edible oil (RBO, SO and RPO) using hexane (25 mg/mL) and subjected to LC-fluorescence (Ex: 298 nm, Em: 325 nm). Toc and T3 were separated by using Inertsil SIL-100A, 3 μm, 4.6 ID × 250 mm (GL Sciences Inc., Tokyo, Japan) set at 40 °C. A solution contained hexane–1,4-dioxane–2-propanol (1,000:40:5, v/v/v) was used as a mobile phase with flow rate 1.0 mL/min. The Toc and T3 concentrations were calculated by using their standard curve (0.1–52.0 ng).

### Statistical analysis

Data are shown as mean ± standard deviation (SD). Differences between groups were determined using One-way analysis of variance (ANOVA) with Tukey post-hoc test. All statistical analyses were performed with IBM-SPSS statistics version 25 (IBM SPSS Inc, Chicago, IL, United States). *p* value less than 0.05 was considered statistically significant.

## Supplementary information

Supplementary information.
