# Supplementary material for: Revealing the thermal oxidation stability and its mechanism of rice bran oil
Source: Sci Rep. 2020 Aug 24;10:14091. doi: 10.1038/s41598-020-71020-y (PMC7445235; doi:10.1038/s41598-020-71020-y)
Supplement: Supplementary file 1 — Supplementary information. [file 41598_2020_71020_MOESM1_ESM.pdf]

Supplementary data

Revealing the thermal oxidation stability and its mechanism of rice bran oil

Halida Rahmania[1], Shunji Kato[1], Kazue Sawada[1,2], Chieko Hayashi[2], Hiroyuki Hashimoto[2], Shigeo Nakajima[2], Yurika Otoki[1], Junya Ito[1], and Kiyotaka Nakagawa[1,*]

[1] Food and Biodynamic Chemistry Laboratory, Graduate School of Agricultural Science, Tohoku University, Sendai, Miyagi 980–8572, Japan

[2] Tsuno Food Industrial Co., Ltd., Ito, Wakayama 649-7194, Japan

[*] Correspondence: kiyotaka.nakagawa.c1@tohoku.ac.jp; Tel.: +81-22-757-4416

(a)

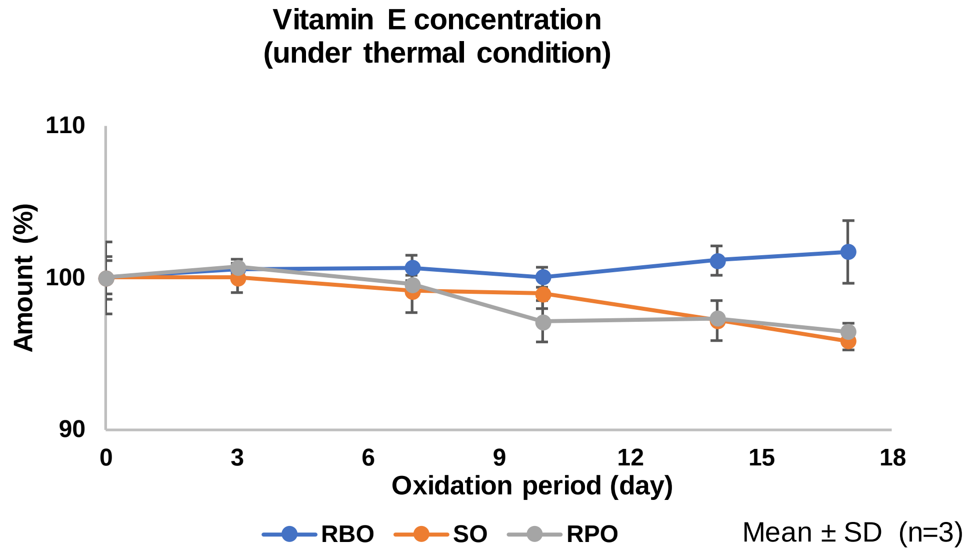

(b)

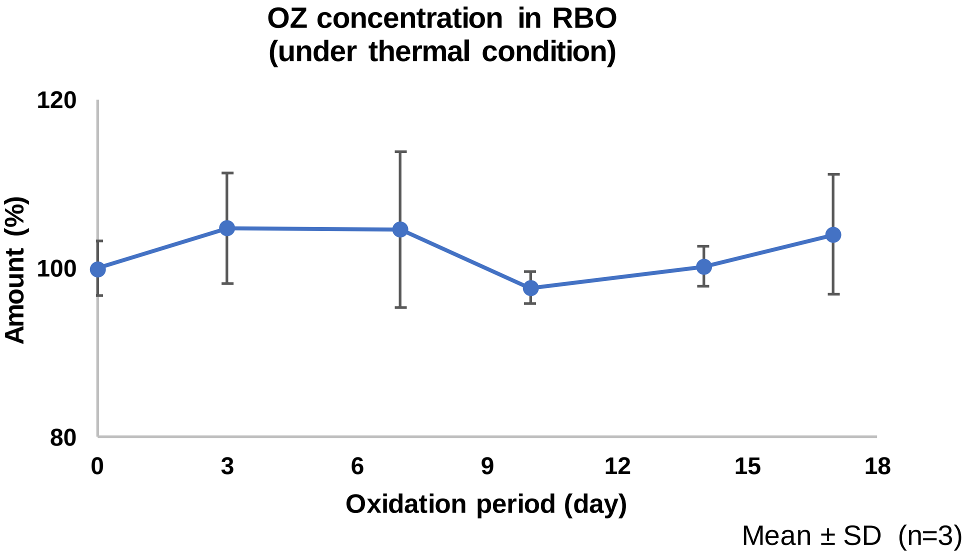

Figure S1. The percentage of amount loss of vitamin E (Toc and T3) in RBO, SO, and RPO (**a**) and OZ in RBO (**b**) under thermal oxidation (at 40°C for 17 days of oxidation period, under dark condition)

Table S1. Fatty acid composition of RBO, SO, and RPO.

	Myristic acid (MA)	Palmitic acid (PA)	Stearic acid (SA)	Oleic acid (OA)	Linoleic acid (LA)	α-Linolenic acid (LnA)
	**C14:0**	**C16:0**	**C18:0**	**C18:1n-9**	**C18:2n-6**	**C18:3n-3**
	**Amount (%)**					
RBO	0.29 ± 0.00	17.42 ± 0.01	1.58 ± 0.01	43.06 ± 0.01	35.90 ± 0.01	0.86 ± 0.01
SO	0.08 ± 0.00	10.99 ± 0.01	3.75 ± 0.01	24.49 ± 0.01	53.72 ± 0.01	5.54 ± 0.01
RPO	-	4.67 ± 0.39	1.71 ± 0.05	64.20 ± 0.22	19.24 ± 0.60	7.00 ± 0.17

Mean ± SD (n = 3)

Table S2. Antioxidant agents contained in RBO, SO, and RPO.

	OZ	Toc (α, β, γ, δ)	T3 (α, β, γ, δ)	Vitamin E (Toc + T3)
	Concentration (mg/100g)			
RBO	129.23 ± 8.15	33.77 ± 0.34	36.71 ± 0.46	70.48 ± 0.77
SO	-	109.40 ± 2.60	-	109.40 ± 2.60
RPO	-	70.96 ± 1.00	-	70.96 ± 1.00

Mean ± SD (n = 3)

Table S3. MS parameter for analyzing TG.

MS parameter	
Polarity	ESI positive
Ion energy	6.2
Collision energy	26
Transfer time	60
Collision RF	1000
Pre pulse storage	10
Capillary	4400
End plate offset	500
Dry gas	1.6
Dry temp.	180

Table S4. MS parameter for analyzing TGOOH.

MS parameter	
Polarity	ESI positive
CUR	20
IS	5500
TEM	600
GS1	40
GS2	80
ihe	ON
CAD	2
DP	126
EP	10

MRM parameter

Product	TGOOH	Q1 (m/z)	Q3 (m/z)	CE (eV)	CXP (V)
HpOTE isomers	OA-LA-(9-HpOTE)	933.9	766.8	37	11
	OA-LA-(12-HpOTE)	933.9	806.8	36	12
	OA-LA-(13-HpOTE)	933.9	847.8	41	21
	OA-LA-(16-HpOTE)	933.9	887.8	38	21
HpODE isomers	OA-OA-(9-HpODE)	937.9	768.9	34	17
	OA-OA-(13-HpODE)	937.9	849.9	38	13
HpOME isomers	OA-OA-(9-HpOME)	939.9	768.9	42	11
	OA-OA-(10-HpOME)	939.9	809.9	51	11
	OA-OA-(8-HpOME)	939.9	754.9	40	10
	OA-OA-(11-HpOME)	939.9	823.9	48	21
